# “Where to find those doctors?” A qualitative study on barriers and facilitators in access to and utilization of health care services by Polish migrants in Norway

**DOI:** 10.1186/s12913-016-1715-9

**Published:** 2016-09-01

**Authors:** Elżbieta Anna Czapka, Mette Sagbakken

**Affiliations:** 1 Sykehuset Innlandet, Norwegian National Advisory Unit on Concurrent, Substance Abuse and Mental Health Disorders, Postboks 104, 2381 Brumunddal, Norway; 2 Norwegian Center for Minority Health Research, Oslo University Hospital HF, Ullevaal, P.O. Box. 4956, Nydalen, 0424 Oslo Norway; 3 Faculty of Health Sciences, Department of Nursing and Health Promotion, Oslo and Akershus University College, Pilestredet 32, 0130 Oslo, Norway

**Keywords:** Polish migrants, Access to health services, Barriers, Facilitators

## Abstract

**Background:**

Poles constitute the largest group of migrants in Norway. Research confirms a steady inflow and a minimal outflow of Polish migrants. One of the key aspects of migrants’ structural integration is access to health care services. This study explored barriers to and facilitators of Polish migrants’ access to Norwegian health care services.

**Methods:**

A qualitative interview-based study was carried out between November 2013 and July 2014. The study is part of a larger, ongoing mixed-method study of Polish migrants’ access to health care services in Norway. Semi-structured interviews were conducted with 19 Polish migrants in Oslo. The interviews were transcribed, coded, and analyzed. Thematic analysis was performed to identify barriers and facilitators related to the use of Norwegian health care services.

**Results:**

Migrants experienced several barriers to and facilitators of access to health care services in Norway. The barriers most often mentioned were problems resulting from insufficient command of the language, related communication problems, and lack of knowledge about navigating the Norwegian health care system. Other barriers related to the organization of the health care system, perceptions of doctors’ skills and practices, and attitudes among health personnel. Factors such as having a Polish social network, meeting friendly health personnel, and perceptions of equal treatment of all patients, facilitated access to and use of health care services.

**Conclusions:**

The study shows that there are both system- and patient-related barriers to and facilitators of migrants’ access to health services in Norway. These findings suggest that successful inclusion of migrants into the Norwegian health system requires regular evaluation of access and utilization of health care services.

**Electronic supplementary material:**

The online version of this article (doi:10.1186/s12913-016-1715-9) contains supplementary material, which is available to authorized users.

## Background

Health care is an important aspect of integration [1], and access to health care services is one element of the structural integration of migrants [2]. Structural integration includes the acquisition of rights and access to positions within the main institutions and arenas in the host society: employment, housing, education, health care, citizenship, and participation in political life [2]. The newest edition of the Migrant Integration Policy Index includes a new health policy strand that enables measurement of health systems’ responsiveness to migrants’ needs [3]. Developing accessible, appropriate, and effective services for migrants poses a huge challenge for health care systems in host countries [1]. Access to health services does not simply mean service availability. According to Penchansky and Thomas [4], access describes the “fit” between patients and health care system. Many governments find constructing new forms of interaction with their citizens difficult and instead rely on existing communication channels. Policy may not match the reality “on the ground”; i.e., entitlement to health care services does not guarantee that migrants have full access to existing services [5].

It has been found that recently arrived migrants are healthier than non-migrants are, which is the so-called “healthy migrant effect” [6]. One of the many explanations for this phenomenon is migrant self-selection; i.e., those who choose to migrate are healthier than average. However, as the duration of the stay abroad increases, the situation reverses because of a series of health risk factors. This contributes to the phenomenon known in the literature as the “exhausted migrant effect” [6]. There is a reason to believe that labor migrants are at risk for deteriorating health. They are prone to stress because of communication difficulties, unfamiliarity with the new environment and culture, and other factors, which may cause serious health problems when combined, especially in migrants with limited access to health care services [7].

Poland’s accession to the European Union in May 2004 generated a marked increase in the migration of Poles to Western European countries [8]. Poles constitute the largest foreign population in Norway, with 91,000 registered Polish migrants as of January 1, 2015, more than a twofold increase from 2009 [9]. Although the instability of modern labor migration makes it difficult to predict the number of migrants who will remain in Norway, there is reason to believe that the outflow of migrants from Norway is small. Research conducted among Polish migrants in 2006 and 2010 indicates that the migrants’ families from Poland often join them in Norway [10]. From a public health perspective, a quick and efficient integration of Polish immigrants into the Norwegian health care system is important. The sooner migrants learn to navigate the Norwegian health care system and the faster and more efficient this system can adapt to the specific needs of migrants, the better for both the migrants and the hosting society [11].

In Norway, it is particularly important that newcomers know how to obtain access to the general practitioner (GP) scheme (*fastlegeordning*). The GP (*fastlege*) is a gatekeeper in the Norwegian health care system and with others expected to provide patients with information regarding specialist medical care. All persons registered in Norway are allocated a personal number and have the right to choose their own GP, and their choice is communicated by post. In case of insufficient knowledge of Norwegian migrants are entitled to an interpreter in medical encounters, a right that is stated in the National Strategy for Immigrant Health [12].

Recent studies of Polish migration to the UK and Norway indicate that migrants are often unaware of the services they are entitled to in the country of settlement [13]. This could result partly from the migrants’ unfamiliarity with the local system and the differences in the organization of health services between the sending and receiving countries. A small-scale quantitative study of Polish migrants’ health-seeking behavior found that Polish migrants use health care services less frequently in Norway than they do in Poland, even when health services are needed [14]. In this particular study, Polish migrants reported several barriers to accessing the health care system [14]. Language-related problems [15–17] and lack of information [15, 18, 19] are the most common barriers mentioned in studies of Polish migrants’ utilization of health services in receiving countries. Further, Polish migrants do not seem to fully trust the health services available and the diagnoses offered by the doctors in the foreign country [15, 19]. Subsequently, patients of Polish origin often consult doctors in Poland instead of or in addition to doctors in their receiving country [18, 20], and they tend to use informal networks instead of professional help for psychological problems [14, 19]. These strategies may reflect a lack of adequate information available for migrants about the range and coverage of health services or their inability to navigate the system.

A review of the literature indicates that there is need for more updated knowledge about how people of Polish origin experience and manage their interactions with health services and their representatives. This qualitative study focus on the barriers and facilitators experienced by post-accession Polish migrants in accessing and utilizing health care services in Norway. The findings are discussed in the light of Kleinman’s concept of “health care systems”: the popular sector (individual, family, and community beliefs/activities); the professional sector (institutional part of health care); and the folk sector (sacred or secular types of folk medicine) [21]. Kleinman conceptualizes illness and disease as socially constructed “explanatory models,” which represent patterns of thoughts that provide answers to questions regarding symptoms, cause, course, and treatment [21]. Thus, the health care system includes people’s beliefs and their behaviors, which to a great extent are governed by cultural rules [21]. The findings are also discussed in relation to transnational theory and the concept of transnational practices [22, 23].

## Methods

Qualitative methodology was used to allow us to explore migrants’ experiences with health care services in Norway. According to the American sociologist Thomas, “if men define situations as real they become real in their consequences” [24]. The action taken by an individual depends on the definition of the situation; therefore, each situation should be studied as it is experienced by an individual.

The study was performed in Norway in 2013. The participants were approached using a semi-structured interview guide (Additional file 1) that allowed the interviewer to tailor the questions to the interviewee and to the interview context. A framework of themes was developed based on the results of previous studies on post-accession migrants’ access to health services in host countries, and was adjusted as a response to the participants throughout the study. Directly after each interview the first author wrote down her first impression and reflections. Before a new interview was conducted, the recordings were listened to and transcribed, followed by a detailed reflection-log, consisting of descriptive and analytical notes. Based on experiences, reflections and identification of novel topics that emerged from the single interviews, adjustments of the interviewing guide were constantly made. Subsequently, some of the themes that seemed important to include were identified before the data production was initiated, while other themes emerged due to the ongoing interviews. The final framework focused on open and overall themes such as barriers and facilitators experienced by Polish migrants when accessing the Norwegian health services and included subthemes such as utilization of health care services in Norway, health information received upon arrival in Norway, migrants’ health information needs, use of interpreter and communication with health personnel. Not all these subthemes are dealt with in this manuscript. The data regarding the two subthemes migrants’ needs for specific health information and migrants’ experiences with interpreter access in medical encounters in Norway will be elaborated elsewhere.

### Sampling method and participants

Interview participants were eight Polish men and 11 Polish women from Oslo and its vicinities. Oslo was chosen because it has the largest population of Polish immigrants. As the health system in Norway is organized in the same way in the whole country, we expected that Polish migrants’ experiences with Norwegian health care services would be quite similar irrespective of the place of residence in the country. The participants were recruited by the first author, who is Polish, has work and family in Oslo, and who often participates in various activities organized by members of Polish community.

A purposeful sampling technique was used aiming at maximum variation [25]. Due to a lack of particular physical arenas facilitating recruitment, the researcher gained access to a variety of informants by the use of three key informants with very different characteristics (e.g. age, education level, work position). These three informants where asked to recruit other migrants with different characteristics (see Table 1) to capture a wide range of perspectives. The recruitment of participants ended when data saturation was reached.

**Table 1:** Sociodemographic characteristics of the participants

	Sex	Age	Education	Self-declared knowledge of Norwegian	Length of stay in Norway (years)
1	M	40–50	Secondary vocational	Basic	7
2	F	30–40	University	Very good	6
3	M	50–60	Basic vocational	Basic	6.5
4	F	30–40	University	Fluent	7
5	F	20–30	University	Basic	1
6	F	30–40	Secondary vocational	Basic	8
7	F	50–60	Secondary vocational	Basic	6
8	M	40–50	Secondary vocational	Good	6
9	F	30–40	University	Basic	5.5
10	F	30–40	Vocational	Basic	7
11	F	30–40	Secondary vocational	Basic	6
12	F	30–40	University	Good	5
13	F	20–30	University	Basic	1
14	M	30–40	University	Good	1.5
15	M	30–40	Secondary vocational	None	2
16	F	30–40	University	Good	6
17	M	30–40	Secondary vocational	None	1
18	M	50–60	Secondary vocational	Very basic	6
19	M	30–40	Secondary vocational	Good	4

The Polish migrants agreed to participate in the interview for a variety of reasons. Some of them said that they wanted to help the researcher and considered the topic to be important. Others hoped to gain some information about how to access and use health services in Norway. Some migrants were particularly interested in the topic because of their extensive experience with health care services in Norway.

All male participants reported migrating to Norway for economic reasons, but most female participants had come to Oslo for family reunification. Our sample comprised of individuals with diversified level of engagement in social activities within the Polish community. Some of the participants were living permanently in Norway with a permanent job, while others went back and forth to Poland and were in a more unstable life-situation. Only two participants declared having a short course in the Norwegian language before they had migrated to Norway. Eight participants reported having only basic knowledge of Norwegian despite the fact that they had spent from 5.5 to 7 years in Norway.

### Data collection and analysis

Interviews lasted for 30–70 min, depending on the participant’s range of experience with the Norwegian health services. Interviews occurred at a place and time convenient for each participant—at home, work, or in a public place. Because of the participants’ long working hours, most interviews took place in the evening or the weekend, and the participants were offered coffee and snacks. All of the interviews were conducted by the first author, in Polish, which enabled the participants to express their opinions in their mother tongue.

Being a migrant herself, the first author is experienced with some of the general problems related to integration into a new society. Moreover, she had an “insider” position by sharing membership with the same ethnic group as the participants [26]. This was valuable during the interview process because the shared experiences functioned as a platform for building trust between the researcher and participants. On the other hand, this could have introduced bias. To avoid reproduction of methodological nationalism [27] and to reduce the influence of assumptions, subjectivities, and prejudices accidentally contributed by the researcher, self-reflexivity was particularly important [28]. During the interviews, the participants and the researcher constantly negotiated their positions. The frequent reference of participants to the researcher’s Polish origin such as “You are Polish, you know how it is in Poland,” is an example of a situation in which the researcher had to encourage the participant to elaborate on a topic that seemed unnecessary to elaborate on because of her position as an “insider” [29]. Another example is when a participant assumed that the researcher was on “their side” because she was Polish. However, it appeared that the informants treated the researcher as an outsider for major parts of the interview because she was living in Norway and carrying out research. The participants asked questions such as “What will result from this research?” and “Why are these interviews necessary for you?”, which indicated that the researcher was also seen as an outsider. During any given interview, the participant’s assumption of the researcher’s position could change several times along the insider–outsider continuum. The fact that the second researcher, who was Norwegian, participated in the whole process of data analysis mitigated the bias that could have been introduced by the “insider” position of the first researcher. The first author interpreted several of the topics based on a Polish frame of reference, while the second author, being Norwegian, would view and interpret the findings based on her experience with the Norwegian society and the Norwegian health care system. Consequently, the analytical process produced data that were a result of a thorough and negotiated version of what the findings represented. We believe this increased the validity of the study as a whole.

Data collection and preliminary analysis alternated constantly during the interviews. All interviews were recorded and transcribed verbatim by the author within 24 h of the time they were conducted, and the important themes were noted. Based on this continuous preliminary analysis, questions were modified and new ones added to the interview guide.

After the data were gathered, they were coded manually, categorized, and analyzed thematically in regards to barriers to and facilitators of access to health services in Norway [30]. The researchers did not disregard existing knowledge but rather built on it by adding new elements and modifying already existing categories (“abductive approach”).

A six-phase approach to thematic analysis was used [31]. In the first phase, the interview transcripts were read three times to obtain a thorough overview of the data (familiarization with the data). In the second phase, the initial codes were assigned to the text. The concepts that the codes represented were inspired mainly by other empirical studies and were modified under the influence of new themes emerging from the interviews. In the third phase, the codes were clustered into themes, both existing and new, that combined codes into categories. In the fourth phase, the initial themes were reviewed to assure coherence of the data within the theme and a clear distinction between the themes. Subsequently, the themes were refined, the data within each theme were analyzed, and subthemes within each theme were identified. For example, in regards to the theme “provision of information,” three subthemes were identified: “letter about the GP scheme,” “health personnel,” and “Internet.” In the last phase, the content of themes and subthemes was merged into generalized descriptions that reflected the significant factors.

## Results

Four main themes emerged from the data: 1) information and knowledge about health care services in Norway, 2) migrants’ experiences related to language competence and utilization of health services, 3) migrants’ perceptions related to the organization and practice of treatment and care and 4) migrants’ experiences with health personnel.

The research findings show that the participants experienced several barriers to access and use of health care services in Norway. The most frequently mentioned barriers resulted from insufficient command of the language and lack of knowledge about how to navigate the Norwegian health care system. Insufficient language competence resulted in difficulties in accessing written information provided in Norwegian, difficulties in navigating Norwegian health related websites, and poor communication with health personnel. Unfamiliarity with the Norwegian health care system, including a lack of understanding of the rationale behind various practices, resulted in lack of confidence in the services. Other barriers were related to health personnel’s attitudes and unexpected costs. Factors that facilitated access and use of health services was having a Polish social network, meeting friendly health personnel, and perceptions of equal treatment of all patients, independent of socio-economic status. The findings will be elaborated further below.

### Information and knowledge about health care services in Norway

When the participants were asked whether and when they had received information about health services in Norway, most of them remembered receiving the letter about the GP scheme (*fastlegeordning*), but they often did not understand the content. Two participants described it as follows:

*I remember that I got a letter probably informing me about how to register to a fastlege (GP). I am not very sure about it because I didn’t understand the language. Maybe there was more information about health care in that letter. I don’t know…I haven’t done anything with that letter. I assumed that I didn’t have any serious health problems. (F2)*

*I got a kind of a letter. As I don’t speak Norwegian, it is hard to guess what it was. (M15)*

Migrants having a poor command of Norwegian used various coping strategies to obtain access to health-related information. Several participants told that one of the most frequent strategies was to ask fellow migrants who speak Norwegian for help. For example, migrants having recently arrived in Norway were bringing letters from the Norwegian Health Economics Administration (HELFO) to Sunday mass and were asking others to translate. Others used colleagues, or people in the Polish community being proficient in Norwegian, to help translate such information letters.

The experiences of the interviewed migrants in relation to their GP as a source of information varied. Some of them were satisfied with the information provided by the doctor. A report of one of the female participants in her forties serves as an example. She received all the required information from her GP when she was considering whether to undergo surgery in Poland or in Norway. Because she spoke little Norwegian, she was always accompanied by an interpreter during her visits to the doctor. On the basis of the information obtained, she decided to have the operation in Norway. Another female migrant, a woman in her 40s, explained how much she liked the letter she received from her GP:

*When I went to my doctor and he sent me the referral to some other doctor, I got the letter and it contained an address, a date, and even a map. I knew where to go, which door, where I was supposed to turn. It is good, I cannot deny this. (F11)*

Even though the letter was in Norwegian, the participant was pleased because the information was so detailed that she felt she did not have to worry about anything. Similar to several of the female participants, her husband, being proficient in Norwegian, helped her with the translation.

Other participants reported that their doctors had not provided relevant information such as information about specialist health care or availability of medical tests when they asked for it. A male informant in his 60s explained:

*When I had to visit a specialist, the doctor told me to choose one. When I got prescribed exercises at a swimming pool, she told me to search for the pool.... And where was I supposed to search? […], I was asking other people, other Poles… (M3)*

The quote illustrates that it can be difficult to navigate the health care system for migrants who speak poor Norwegian, are not familiar with the Norwegian society, and whose only source of information is other Poles. As the findings show, for many of the participants, the main source of information about health care in Norway was other Poles, especially those who are experienced in using health care in Norway. For the female participants, who had come to Norway for family reunification, the most important source of information was their previously arrived husbands.

Some of the participants said that their GP referred to the Internet as a source of information. However, because of several barriers including poor computer literacy and lack of knowledge of Norwegian, many had limited access or limited understanding of the health information available on the Internet. Several told that they found Norwegian health-related websites rather difficult to access and navigate, and was too complicated to use.

Some participants emphasized that health related websites in Norway lack English links and that there is a lack of online brochures in Polish and English. This makes it difficult for migrants who do not speak the language to use relevant websites or read relevant brochures. A female participant in her 30s searched for information relating to ways of dealing with a specific medical problem in Polish and English websites. She explained:

*I was also looking for it in other countries, in Great Britain and Poland. It is not always the best strategy because in other countries, there are different approaches… but at least, thanks to the Internet, I could deal with it somehow, with this information gap. (F13)*

This participant was aware of the problems she might encounter using a website representing another country. Although the coping strategy she used was not ideal, it helped her to fill the information gap.

The findings also show that one of the barriers in accessing health services related to a lack of understanding of the way of organizing Norwegian health services, including the rationale behind different referral and treatment practices. A man in his 50s elaborated:

*Until today I don’t even know what tests I am entitled to, what I can ask for, what examinations the doctor can refer me for… if I can ask the doctor for it, or if I can go to see some other doctor. I know from my personal experience that the doctor who treats me approaches it [disease] very reluctantly. He seems to think that he is treating me, but after the consultations with Polish doctors it turns out that he should have directed me here or there.... Because of my own security and lack of doctors’ knowledge here, I consulted doctors in Poland. (M8)*

Despite declaring a good command of Norwegian, this participant decided to consult doctors in Poland. The decision was based on a general sense of insecurity about the Norwegian health care system, his experience with Norwegian doctors, as well as the different treatment and referral practices in the Norwegian and Polish health care system. Narratives provided by other participants indicates that new-coming migrants have low level of health information literacy due to subtle differences between Norwegian and Polish health care systems. A man in his 50s elaborates:

*People who have been here long enough know how to do it in both systems, where to go, where and how to find doctors. The same as I know what to do in Poland. I haven’t grown up in Norwegian system so I must learn it now. (M13)*

The participant emphasises that it is particularly difficult to navigate the new health system in the beginning, but that after a while migrants develop a double competence in which enables them to use health care services in both countries.

### Language competence and interaction with health personnel and health services

All participants mentioned language as one of the main barriers to their access to health services. A woman in her 40s explained why the mutual understanding between a doctor and a patient is vital:

*Well, certainly it would be better for foreigners if there were the possibility of common understanding. So that they explained everything in such a language that you understand. It is not like buying bread—it is your health. If you do not understand something, it can be dangerous. (F12)*

Most of the participants emphasized that the language barrier is the biggest problem for newcomers and that those that do not speak the language are the ones who require the most help from the host country. One of the participants became pregnant after her arrival in Norway. Because of insufficient command of Norwegian, she was afraid to visit a Norwegian doctor:

*In the end, I have tried so long for this child. I wanted everything to be okay. My first concern was the fact that I had a Norwegian doctor and I didn’t know how to communicate with him. After all, I didn’t know the language, did I? It was horrifying for me, such a terrible fear. (F10)*

This quote shows how migrants can feel unsafe and frightened about their interactions with a doctor who do not speak a familiar language at a time where they want to feel safe and cared for.

Although most of the participants used the help of other Polish Norwegian-speaking migrants, some tried to cope on their own. A case of a young man who had a car accident in Norway and required long-term treatment is one example. He bought himself a medical dictionary to be able to prepare an appropriate medical vocabulary for his visits to the doctor. Others noted that they used the Google Translate application to translate the letters from the HELFO or the information from the HELFO’s website.

For some participants, the language barrier turned out to be insurmountable. A woman in her 40s, who did not speak Norwegian and whose husband used to translate for her during the visits at the doctors, told how she stopped visiting doctors in Norway:

*I don’t go to Norwegian doctors anymore to make it easier for me and for my husband. (F11)*

This quote illustrates a change in behavior that was common for many of the participants. After using Norwegian doctors for some time, the strategy changed because of experiences with misunderstandings or a feeling of not being understood. A woman in her 30s, who had been living in Norway for a year and declared a basic knowledge of Norwegian, explained why she preferred using health services in Poland:

*In Poland, however, I think I feel more secure at the doctor’s in terms of linguistic competence. I understand what everybody says to me. (F5)*

Others claimed that the use of Polish health services by Polish migrants was partly a result of language barriers and partly a result of overreliance and trust in a system they already knew. In some cases, this lead to what may be seen as irrational practices, such as one of the participants calling Polish acute lines or Polish hospitals when becoming ill in Norway. However, for many of these participants, discussing a problem in a familiar language seemed to be a better and safer option than presenting it in another language at a local hospital or doctor’s office.

During the interviews, the participants repeatedly commented on the different kinds of barriers to accessing an interpreter. A woman in her 40s noted the inconsistency in accessing an interpreter based on the experiences of a close friend:

*She [a friend] had to arrange for the interpreter herself. This is how it is here. Sometimes they provided her with an interpreter and other times they would say that they are very sorry, but they couldn’t get her an interpreter because it is too expensive for them to organize one and she has to bring her own interpreter along to the visit. (F6)*

However, most participants considered being offered an interpreter’s help as very positive. One of the participants emphasized that the assistance of an interpreter when visiting a doctor was needed mainly at the beginning of somebody’s stay in Norway; when they did not know the language yet and had to learn how to cope in a new country.

One participant, a woman in her 30s, was skeptical about the right to use the free services of an interpreter by immigrants. She perceived it as something that could have negative consequences in the long term:

*Because it is what the Norwegian system does. It allows people to function over a long time without the knowledge of the language…It does a bit of disservice to those people. (F4)*

This participant stated her view that, to motivate migrants to learn the language, the interpreter’s services should be paid by the patient after the first year of residence in Norway.

### Migrants’ perceptions related to the organization and practice of treatment and are

The question of payment for the use of health services came up frequently in the interviews. Not everyone was aware that the use of medical services in Norway requires a personal contribution toward the cost of treatment (*egenandel*). This was illustrated by the comments of a woman in her 30s and a man in his 50s:

*For Poles, that patient fee is high. (F5)*

*I always thought that if one works, health care is free of charge in Norway. However, when I went to a doctor for the first time, I was very surprised because I had to pay something. (M8)*

Although the *egenandel* is a relatively small cost, it was described as surprising and an economic barrier among the newly arrived migrants with dependent family members in Poland.

Many participants stated that another reason why they preferred to use health services in Poland was that in Norway GPs “treat all diseases” (M3), which they thought affects the quality of the doctors’ work. Some participants declared that this practice was the main reason why they visited medical specialists in Poland. A female participant in her 30s explains why Polish patients have limited confidence in the GPs in Norway:

*Here, you go to a GP with all your health problems. We don’t really like it. I think that a GP doesn’t have such an extensive knowledge as a cardiologist, for example, a specialist who deals especially with the heart and its diseases. I think, that a GP will not advise me just like a cardiologist. Even if he studied it, his knowledge is not just like focusing on cardiologic specialization. (F12)*

This quote illustrates how people from Poland may be both unfamiliar and uncomfortable with the Norwegian GP’s treatment of a broad spectrum of diseases. Their experiences from Poland differed in the sense that a referral to a specialist was more common after diagnosis or during the diagnostic process. This seemed to cause them to distrust the extensive treatment provided by GPs in Norway.

Another barrier related to the organization of health care was that Polish people who come to Norway for up to 6 months obtain only a temporary personal number (D number). The participants interpreted this to mean that they were in a specific situation and it caused insecurity whether they had the right to choose a GP, and if not, where to get help when they were ill. One of the participants, a man in his 40s, had been in such a situation before he received a national identity number:

*In Norway there is still such a thing that if we have a temporary ID number, we don’t have this ‘fast’ [GP], so I guess, we need to deal with it on our own. I don’t know how it is. (M19)*

The quote exemplifies a general lack of knowledge among migrants with a D number about their entitlement to health care in Norway, which was the case for several of the participants.

The findings also indicate that particular elements of the organization of health services in Norway facilitated the participants’ access to health services. For example, a woman in her 30s who had a child appreciated the appointment reminders:

*I like it here, that in case of children’s vaccinations or appointment, you always get a reminder. (F12)*

Some participants mentioned the efficient organization of visits at health centers. A man who had been living in Norway for 4 years explained the differences between the organization of visits to doctors in Norway and in Poland:

*If I have an appointment with a doctor at 9 o’clock and I will come at 9, I already know that this time will be dedicated only for me. In Poland, we do not have such freedom to determine the time of appointment, and we often queue in front of a doctor’s surgery. (M19)*

The quote shows that Polish migrants appreciate being able to schedule a visit to a doctor and that they are confident that the doctor will see them at the appointed time.

One of the participants, who had been residing in Norway for 7 years, raised the issue of bribery. In Poland, giving bribes to doctors, although illegal, was described as quite common. A female participant in her 40s reflected on the consequences of the inability to offer bribes to Norwegian doctors:

*Here you don’t need to give a bribe to your doctor because most doctors earn a lot. You don’t have to pay them in order to get care. Maybe it is easier in our country because if you pay, you can speed up the process. Here there is no such possibility. Still, I think it is really good that one doesn’t have to give bribes. All patients are treated equally here. You do not have to pay them to take care of you. (F4)*

Overall, this participant considered the lack of the need for bribes a positive feature of the Norwegian health care system because it ensures equal treatment of all patients, regardless of their financial situation. Some of the other participants also mentioned that they appreciated this type of “equal treatment”. For instance, a woman in her 40s stated that the medical staff “treats everybody the same,” implying that the doctors and nurses would behave in the same way independent of socioeconomic status, and subsequently also the ability to offer bribes.

### Migrants’ experiences with health personnel

Some participants, especially women, claimed that they felt disregarded by the doctors merely for being migrants. For example, two young, highly educated female migrants who had given birth in Norway described what they saw as different treatment related to guidance on breastfeeding. One of them, a woman in her 40s, described this as follows:

*I heard many stories from Polish people. First of all, they feel that they are disregarded. Many people have the impression that doctors have different attitudes toward a foreign patient… I came to that impression at the time when I was in the delivery room. My daughter was born and I had problems with breastfeeding. A nurse, a specialist in breastfeeding, told me that it would not be a problem if I didn’t breastfeed my baby. It is not a crisis. I had a feeling that if I had been a Norwegian woman, she would have tried harder. (F4)*

This quote shows that the participant felt disregarded because, in her opinion, she was treated differently from Norwegian patients. The woman knew that in Norway great significance is placed upon breastfeeding, and she felt discriminated against because this was not emphasized as something important. The other woman described how the nurse treated her in an “insensitive way” when she could not breastfeed, and she interpreted the nurse’s behavior toward her as different compared with the Norwegian mothers.

A female participant in her 30s suggested that a feeling of being discriminated against might result from lack of knowledge about the function of the Norwegian health care system:

*I think that Poles often believe they are being discriminated against when they are treated like any Norwegian patient, but differently than in Poland. (F13)*

There were also examples of participants that described being treated differently by the doctors in Norway as something positive. A man in his fifties, with vocational education, receiving rehabilitation benefits (money and training program) for 6 years, provided an example of a positive experience:

*Here, in Norway, the doctors treat us just like in Polish private clinics. Just like that. It’s a great convenience. In Poland, when you go for private care, you will receive the same treatment as in Norway… Here, doctors approach a man as a human being. Here, you talk as equals. There is respect for people. It is a big difference between Poland and Norway. (M3)*

As in many of the examples, the participant compares and explains his experiences from the country of origin when evaluating the health services in the host country.

Another issue that was raised by most of the participants was related to doctors’ treatment and prescription practices in relation to a variety of conditions. Many complained about the fact that doctors prescribed them only mild painkillers, as observed by a well-educated woman in her 30s:

*If they don’t want to prescribe an antibiotic, they should prescribe at least some ointment. Whatever. They have only two recommendations, paracet or staying at home and you will recover. (F12)*

The quote shows that some Polish migrants equate serious treatment by a physician with a prescription. Offering paracetamol as the only drug followed by the recommendation to rest may provoke a sense of discrimination and give rise to a lack of confidence in Norwegian doctors.

Several women also expressed their dissatisfaction with the fact that doctors do not want to prescribe drugs, in particular antibiotics, to children. However, many of those who had been in Norway for many years had become used to the different methods of treatment, including less frequent use of antibiotics. One of the female migrants in her 30s, who had two children in Norway said:

*When I came to Norway, it scared me. My God, the baby is sick and they do not give antibiotics, they don’t give anything. I used to think in Polish. As I went with my daughter to the doctor in Poland, he examined her rather briefly and immediately prescribed her an antibiotic. And here these children are not given any medication. How to live here, nothing but die. But I got used to it and I even like it. (F6)*

The quote describes the change in mindset about the frequent use of antibiotics by children. The participant acknowledged that the use of antibiotics by her child in Poland might have been unnecessary and, now that she understood the rationale, she valued the limited prescription of antibiotics among Norwegian doctors.

One of the participants, a woman in her 40s with higher education, mentioned another reason why Polish people sometimes feel discontented when meeting with Norwegian doctors. According to her, Polish people tend to see the doctor only in a situation where they think medical assistance is strictly necessary. Consequently, doctors in Norway should treat Polish migrants more seriously:

*You know, we Poles usually go to the doctor when there is really something serious. We don’t go to see the doctor with just anything. This mere fact should be a warning sign to the doctor that this person really needs help. (F9)*

The quote refers to the perceived prevalent practice of telling patients to rest or to take painkillers; in other words, they feel they are not being taken seriously as a patient in need of medical care.

Many of the interviewed migrants, especially men, stressed the immense kindness of Norwegian medical personnel toward patients. In particular, several noted that the nurses in Norway were always smiling. A man in his 40s who used the health services regularly because of an accident stated:

*Everyone was super nice and polite. Especially nurses. I got the impression that I ended up in a medical series. (M14)*

Several participants emphasized that the friendly attitude from the medical staff toward migrant patients was particularly important, and experience of friendly attitudes seemed to counteract feelings of discrimination.

The participants did however often make negative statements about the competence of Norwegian doctors. According to one of the participants, the doctors lacked sufficient medical knowledge because, during a visit, “he [the doctor] took out the book and switched on the computer*”* (F10). Several people mentioned that the doctors in Norway have high-quality equipment, but they do not know how to use it or how to read the results of the tests. A woman in her 40s who has lived for 8 years in Norway and has frequent contact with Norwegian medical personnel explained:

*In general, they have very good equipment. But there comes an examination result and they are not able to read it. Norway lacks qualified people. At least I have that impression.... In Poland, the opposite is true. There are qualified people who cannot demonstrate their competence… (F6)*

This quotes illustrates how migrants may view the doctors’ use of resources, such as the National Treatment Guidelines for Health Personnel (*legemiddelhåndboka*) or Internet (e.g., searching on the Internet when interpreting test results or looking for relevant medication) in front of the patient, as a sign of the doctor’s insecurity and incompetence. The quote also shows that, despite the high quality of the equipment used by Norwegian doctors, Polish migrants tend to rely more on doctors who openly show their experience and security when interpreting test results and providing treatment.

## Discussion

The aim of this study was to identify the main barriers and facilitators experienced by post-accession Polish migrants in accessing and utilizing health care services in Norway. The findings of this study are also relevant for understanding other migrant groups’ experiences and perceptions of the health care system.

In this study, we found that one of the main barriers in access to health services in Norway was lack of sufficient information about the organization of the health care system. The health care system in Poland was an obvious point of reference for most of the participants. The differences between Polish and Norwegian health care systems seemed to represent the basis on which both positive and negative conclusions were drawn. A study of immigrants in the Netherlands found that (ongoing) contact with the health care system of the country of origin greatly influenced the perceptions of various dimensions of the Dutch health care system [32] and the expectations about the GP’s means of providing services. Similarly, a qualitative study of 13 migrant groups in Norway (including Poles) found that conflicting ideas about the role of the doctor, language proficiency, and an inadequate comprehension of the Norwegian health care system represented barriers to accessing both GPs and other parts of the Norwegian health care system [33]. Interestingly, another qualitative study from Norway including GPs, showed that they had similar opinions on migrants’ barriers in access to health services. Migrants’ difficulties in accessing and handling Norwegian health services was explained as resulting from insufficient language competence and different expectations or conflicting understandings of the doctor’s role. The GP’s described difficulties related to expectations in regards to the doctor’s authority, as well as the power of medicine and the ability to restore health. Further, the GP’s expressed a preference for an interaction where patients see themselves as contributors to their own health in which includes an emphasize on preventive measures taken by the patients [34]. This dialogue-based model, focusing on patient’s own responsibility, may be very different from the doctor-patient interactions as experienced in Poland.

A review of the potential barriers to the use of health services among ethnic minorities show that communication styles and attitudes among health personnel are the main barriers to accessing quality services. In addition, organizational aspects of the health care systems, such as differences in the referral system, represent major barriers as perceived by people with experience of other health care systems [35]. In our study, differences in treatment approaches represented a significant barrier to health service utilization and patient satisfaction. In particular, the participants emphasized the reluctance of Norwegian doctors to prescribe antibiotics and that doctors tended to suggest rest and paracetamol as treatment instead of offering what they considered “real” treatment, such as other types of medicines and/or further examinations. Similar findings are described in studies of post-accession Polish migrants in Great Britain [18, 35].

The participants in our study also seemed unfamiliar with, and did not appreciate, the Norwegian GPs’ approach to and treatment of a broad spectrum of diseases. Their experiences from Poland differed in the sense that there are more referrals to a specialist after diagnosis or during the diagnostic process and this comparison seemed to cause a general unease and distrust of GPs in Norway. Unease or distrust seemed to be strengthened by visits to doctors who searched for information in the Norwegian Pharmaceutical Product Compendium (*Felleskatalogen*) or the Internet; a finding that has been reported by a previous study of immigrants in Norway [33].

In the process of migration and subsequent socialization, an individual gradually internalizes a system of symbolic meanings, values and standards, governing behavior as well as certain ways of perceiving the world ([21], p.36). In addition, people acquire specific patterns of communication in regards to people belonging to the new community. Therefore, migrants, accustomed to a particular health care system and its associated meanings, values, and standards need to learn how health care is organized in the country of immigration and the rationale and values that underlie this organization. According to Ingleby, immigrants have to “learn a lot more than where to find the waiting room or how many times a day to swallow their pills” ([36], p. 23). Migrants from Poland may not be aware of the role and function of GPs in the health care system. Norway has a tax-financed public health care system, and the GP acts as a gatekeeper to secondary care (hospital care being free of charge). GPs, many of whom hold a specialty in General Practice, are expected to use their best professional judgement to secure effective and fair allocation of resources, which implies that they must allocate resources between patients with competing needs [37]. For decision making within health care, this signifies that any use of health care resources may be seen as denying the opportunity for another patient to use the money for potentially greater benefit [38]. In Poland, the system is based on a general insurance model, and health care is provided free of charge to any person with health insurance. GPs in Poland act as a gatekeeper to secondary care, with some exceptions, such as specialized clinics in gynecology–obstetrics, ophthalmology, oncology, psychiatry, dentistry, and sexually transmitted diseases, which can be accessed directly. These different models of health care, with their implicit values and norms, may explain the unfamiliarity and unease among Polish migrants in regards to the GPs’ treatment practice and referral routines. The models signify a complexity of politics and values that may be unfamiliar or not reflected on by the participants. Many Polish migrants receive information about the professional part of the health care system from a broad range of representatives (e.g., family, friends, and colleagues) [21], which may allow misconceptions to be established, which are not modified later.

Another barrier related to the organization of health care was that Polish people who come to Norway for up to 6 months only receive a temporary personal number (D number). This implies that they are in a specific situation and have no right to choose a GP. They have the right to health care in case of illness but may not know where to get help when ill. A small-scale quantitative study conducted in Norway shows that Poles possess only rudimentary knowledge of the GP scheme and that, as a result, they do not know how to access health services [14].

Even though the maximum fee to be paid for health services is 230 euros per year (excluding dental care), the participants considered this as a significant obstacle to using health services. These findings are consistent with those of an earlier study from Norway [14]. Studies from Great Britain have shown that Polish migrants complain about the high cost of medical services, especially dental care [20, 39]. In addition to reflecting the participants’ economic situation, this may also indicate that migrants may not know about the upper payment limit for the use of health care services in Norway. In Poland, the health care system is insurance based, and statutory health insurance is compulsory for employees, the self-employed, people working in state education, and people on government benefits or pensions; the insurance scheme covering 98 % of the population [40]. In most cases, health insurance dues are paid by the employer or other institutions that pay benefits (e.g., the Labour Office for unemployed people). This implies that, for most of the population in Poland, health services, including dental treatment, are free of charge.

The findings of our study also indicate that certain elements of the Norwegian health care system may act as facilitators to accessing health care services. According to the participants, several undesirable phenomena that are characteristic of the organization of the health care system in Poland are absent. The examples given were the extra costs of bribery and having to queue outside the doctors’ offices, which makes it difficult to plan the duration of an appointment. In addition, several participants emphasized what they described as the “equal treatment” of all patients, which seemed to describe a friendly, modern, and egalitarian attitude among health workers. A review of barriers experienced by minority groups’ when using health care services noted that verbal and nonverbal communication styles and different factors related to the general patient approach are all important to avoid feelings of discrimination, racism, and stigmatization [35]. As shown in our study, even subtle feelings of being treated differently than the majority population may evoke emotional distress among minorities.

Acculturation into a new health care system is a complex process. Because of specific barriers or facilitators, it may be accompanied by varying degrees of perceived access and satisfaction with existing services. The results of our study indicate that problems with access to adjusted information constitute one of the main barriers to Polish migrants accessing quality health care in Norway. Despite the use by many of the Internet to find information about the Norwegian health care system, this is inadequate in the case of migrants who are unfamiliar or not proficient with the Norwegian language. The availability of Internet pages and brochures in Polish and English is relatively limited. The difficulties in accessing adjusted information are in line with earlier studies conducted in countries receiving post accession Polish migrants [15, 18, 19, 39, 41], including Norway [14].

In general, the lack of language competence or adjusted information are two of the most common barriers quoted in studies of migrants’ access to health services in EU countries [42]. These factors are also among the most commonly cited factors in a review of barriers to the use of health services among ethnic minorities [35]. These findings are also consistent with several primary studies conducted in other receiving countries [15–17, 43, 44]. For example, a study from the Czech Republic found that the use of GPs by different migrant groups was positively correlated with their knowledge of the language [45]. A cross-sectional study from Greece documented how migrants’ increased knowledge of existing health services was associated with increased language competence [46]. Similarly, health care professionals in 16 European states describe barriers related to language and the ability to provide adjusted information as profound in the meeting with migrant populations [47]. Language barriers include access to and use of interpreters. Our study identified several barriers associated with access to interpreting services, although for patients lacking competence in Norwegian, health personal are obliged to ensure that the patient has understood the content and significance of the information [48].

As Kleinman [21] note, health personnel may not necessarily consider their patients’ experiences, beliefs, and values when providing information. Subsequently, they may not be aware that health-seeking behaviors that may seem different — even irrational or inconsistent — may arise from migrants’ exposure to a health care model or “explanatory model” ([21], p. 58) entailing different ideas, values, and priorities. Lack of such knowledge on both sides may cause distrust between patients and representatives of the health care system. Migrants may feel disregarded and discriminated against when, in fact, they may be treated in the same way as the inhabitants of the receiving country. In addition to migrants’ experience of other barriers, such a sense of discrimination may cause them to avoid using Norwegian health care services.

Both our study and previous studies in Norway, Great Britain, Germany, and Spain [14, 18, 20, 49] indicate a common transnational use of health services by post-accession Polish migrants. When using the transnational lens to analyze migrants’ activities, one needs to let go of the conviction that social life takes place naturally within the nation-state. Migrants are involved in transnational practices because they are located within transnational social fields [22, 23]. The findings in this particular context indicate that it is common to assess the Norwegian health care system and its representatives through the prism of experiences of the Polish health care system. However, transnational practices may mean that migrants use public, primary health services while they are in Norway, but use specialized doctors in the private sector while they are in Poland. In the same way, they may also change or adjust socially constructed explanatory models, which represent patterns of thoughts that provide answers to questions regarding symptoms, cause, course, and necessary treatment [21]. Thus, a person’s health-seeking behaviors may change and adapt over time and in accordance with the cultural understandings and practices existing in a given context at a given time. The findings of this study shed light on important contextual and structural barriers, independent of whether they are “real.” Illuminating both the barriers and facilitators may help create more effective interactions between the Norwegian health care system and the largest immigrant group in Norway.

### Study strengths and limitations

The strength of this study is its relevance. We have focused on Polish migrants, the largest migrant group in Norway, which is expected to continue to increase in Norway. To facilitate their integration into the health system, it is necessary to identify the barriers and facilitators they experience in accessing health care services. The main weakness of the study was the sample size. Although we believe that we reached data saturation in our sample, we are aware that other migrants may have different experiences with the Norwegian health system. The fact that the position of the researcher often changed during the interviews along the insider–outsider continuum was one of the challenges of the study. This position may be seen as both a strength and weakness. However, the second author is born in Norway, and the analysis and discussion of the findings represent a negotiated construction of the empirical data, which we consider as a strength.

## Conclusions

This study was conducted to identify the main barriers and facilitators experienced by post-accession Polish migrants in accessing and utilizing health care services in Norway. However, the study is also relevant for understanding other migrant groups’ perceptions and experiences of health care systems. The findings suggest that barriers to and facilitators in accessing health services in Norway are associated with both system- and patient-related factors. Patient-related factors relate to language competence, knowledge about the health care system, and understanding and preferences related to explanatory models and practices in the host country. System-related factors relate to the qualities of the health care system the migrants encounter, unexpected costs, health personnel’s attitudes and practices, provision of adjusted health information, and access to the GP scheme. The findings in this study suggest that successful inclusion of migrants into the Norwegian health care system may need regular evaluation of access and utilization of health care services.

## Additional file

Additional file 1: Interview guide. (DOCX 14 kb)

## Abbreviations

GP: General practitioner
HELFO: Norwegian health economics administration
REK: Regional committees for medical and health research ethics
