# Supplementary material for: “Where to find those doctors?” A qualitative study on barriers and facilitators in access to and utilization of health care services by Polish migrants in Norway
Source: BMC Health Serv Res. 2016 Sep 1;16(1):460. doi: 10.1186/s12913-016-1715-9 (PMC5007991; doi:10.1186/s12913-016-1715-9)
Supplement: Additional file 1: — Interview guide. (DOCX 14 kb) [file 12913_2016_1715_MOESM1_ESM.docx]

**Interview guide**

The interview begins with providing information about the project and interview aims, research/interview process and research ethics.

1. First, I would like to ask you a few questions about yourself.

How long have you been in Norway? Why did you come here? Do you like being here?

How old are you? Could you tell me about your background and education?

1. What do you usually do when you feel sick in Poland? Where do you look for help?

Have you had any health problems while being in Norway? Have you had any new problems since you came to Norway? Where do you look for help when you feel sick? How do you deal with it?

1. Did you receive any information about the health care system in Norway when you came here? If so, what type of information? How did you receive it? From whom (healthcare, employer, church, caritas, NAV, internet, friends, family)? In what language?
   What do you think of information you receive via post (for example letter about GP scheme in Norway)?
2. Do you have any difficulties obtaining information about the Norwegian health care system? Where do you look for such information? What kind of information do you need? What form or type of information would you prefer to receive? Would you like to obtain information about specific health problems? What problems?
   In your view, what information would be important for newly-come immigrants?
3. Have you used the Norwegian health care system (GP, nurse, dentist)?

Have you had any problems accessing the doctor? What sort of problems?

Have you encountered any help accessing the GP? What have caused (potential) problems using the Norwegian health care system?

What positive impressions do you have concerning the organisation of the Norwegian health care system? Examples?

What positive impressions do you have concerning the use of the Norwegian health care services? Examples?
